# Dynamics of large pyroclastic currents inferred by the internal architecture of the Campanian Ignimbrite

**DOI:** 10.1038/s41598-020-79164-7

**Published:** 2020-12-17

**Authors:** Claudio Scarpati, Domenico Sparice, Annamaria Perrotta

**Affiliations:** Department of Earth, Environmental and Resources Sciences, University of Napoli Federico II, 80126 Napoli, Italy

**Keywords:** Natural hazards, Sedimentology, Volcanology

## Abstract

Large ignimbrites are the product of devastating explosive eruptions that have repeatedly impacted climate and life on global scale. The assemblage of vertical and lateral lithofacies variations within an ignimbrite sheet, its internal architecture, may help to determine how the parental pyroclastic current evolves in time and space. The 39 ka Campanian Ignimbrite eruption, vented from Campi Flegrei caldera, laid down a thick ignimbrite over an area of thousands of km^2^. A detailed reconstruction of the vertical and lateral variation of the seven lithofacies recognised in the ignimbrite medial sequence constrains the behaviour of this event. The pyroclastic current flowed over a wide area around Campi Flegrei without depositing (bypass zone), and inundated a huge area during most of the paroxysmal, waxing phase, emplacing a mainly incipiently- to strongly- welded ignimbrite. Following this waxing phase, the leading edge of the current retreated back towards the source as the current waned, impacting a progressively smaller area and leaving an unconsolidated ash and lapilli deposit, later lithified. Our study illustrates how large pyroclastic currents can evolve in time and space and the importance of both internal (eruptive and transport mechanisms) and external (topography, surficial water and rain) factors in governing their behaviour.

## Introduction

**Figure 1:**
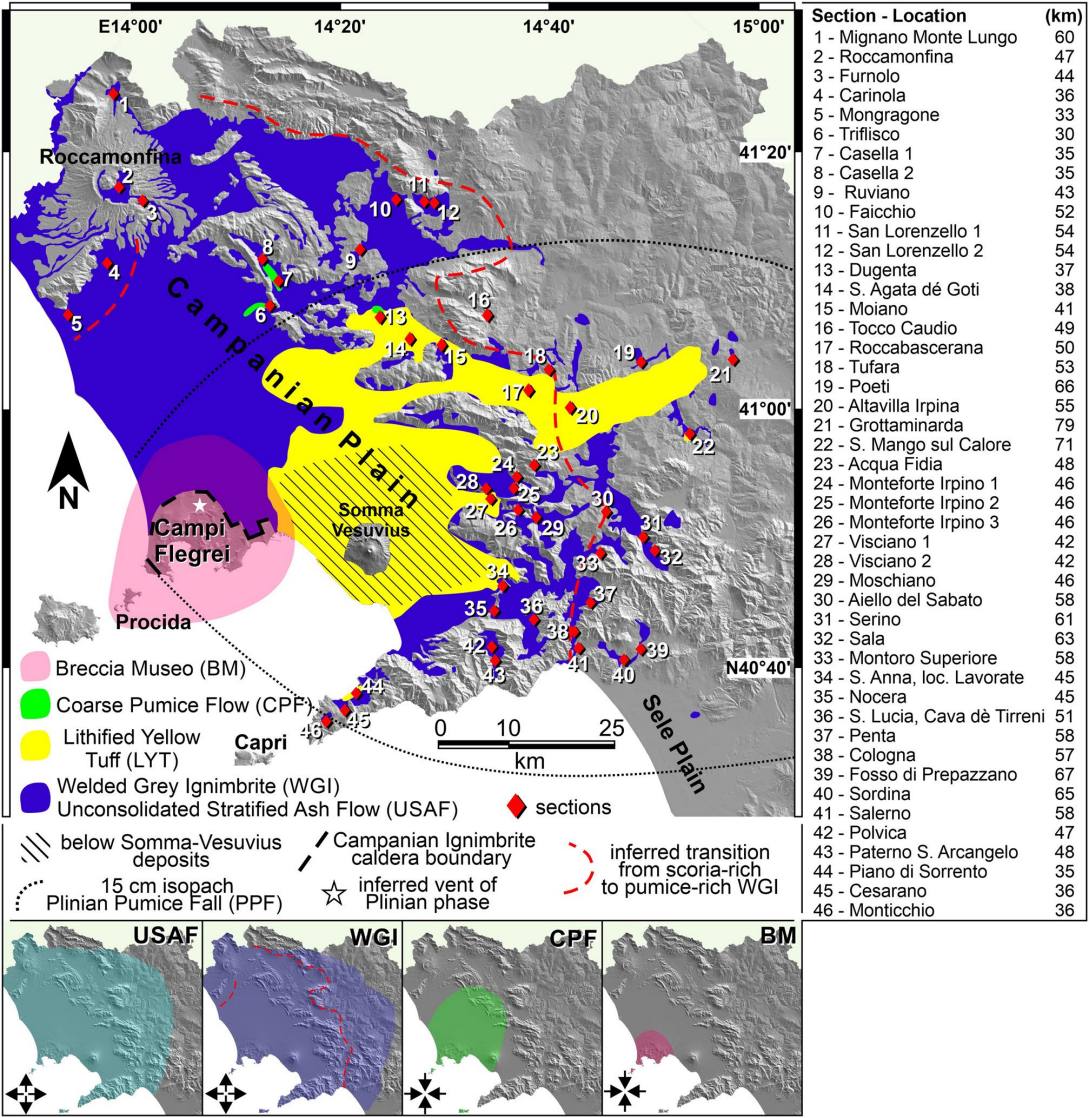
Campanian Ignimbrite pyroclastic current deposits distribution^13–17^ and inferred vent location of the Plinian phase^18^. The unconsolidated stratified ash flow deposit is ubiquitously underlying welded grey ignimbrite. Inset: spread of the CI pyroclastic current at different times; the inundation area increases (outward-pointing arrows) as the current waxes to a climactic phase (welded grey ignimbrite) and then decreases (inward-pointing arrows) as the current wanes (coarse pumice flow deposit and Breccia Museo). Location and distance from source of studied sections is listed on the right. Figure is generated using CorelDRAW Graphics Suite 2020 for Windows v. 22.0.0.412 (https://www.coreldraw.com/it/product/coreldraw/).

**Figure 2:**
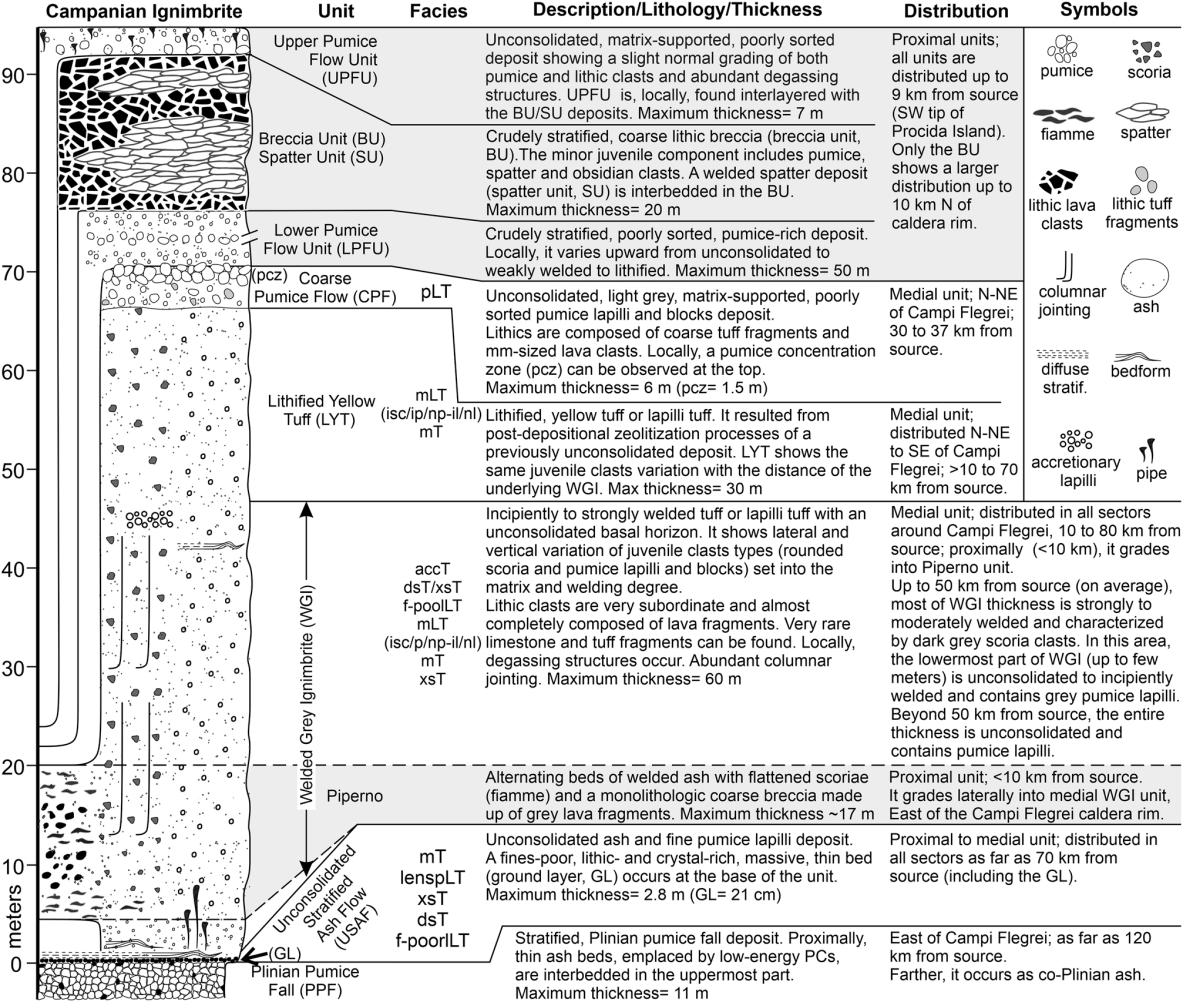
Composed stratigraphic section of the Campanian Ignimbrite with summary of the main characteristics of the recognized units. Grey shaded area (plus the ubiquitous unconsolidated stratified ash flow deposit) indicates the units forming the proximal Breccia Museo. Facies abbreviation: f-poorlLT (fines-poor lithic lapilli tuff); mT or mLT (massive tuff or massive lapilli tuff), isc/ip (inverse graded scoria/pumice), np (normal graded pumice), il/nl (inverse/normal graded lithic); dsT (diffuse stratified tuff); xsT (cross stratified tuff), pLT (pumice lapilli tuff); lenspLT (lenses of pumice lapilli tuff); accT (accretionary lapilli-bearing). Figure is generated using CorelDRAW Graphics Suite 2020 for Windows v. 22.0.0.412 (https://www.coreldraw.com/it/product/coreldraw/).

Catastrophic pyroclastic currents impact huge regions and represent one of the most devastating natural phenomena^1^. Large pyroclastic currents emplace thick sequences of ash- and vesiculated juvenile-rich deposits (ignimbrite^2,3^). Large ignimbrites show changes in facies on a regional scale^4^. There are only a few large ignimbrites that have been subject to detailed studies of their three-dimensional facies architecture; these include the Bishop Tuff^5^, Taupo ignimbrite^6^, Novarupta ignimbrite^7^, Cerro Galán ignimbrite^8^, and Neapolitan Yellow Tuff^9^. In order to broaden our understanding of these eruptive events, studies of additional, well-exposed examples are needed. Here, we present a detailed examination of the medial pyroclastic current deposits (10 to 80 km from source) of the Campanian Ignimbrite eruption (CI), a caldera-forming Plinian event occurred 39 ka ago^10,11^, whose pyroclastic current spread over a huge area from Campi Flegrei (Italy) emplacing a thick ignimbrite sequence (Figs. 1, 2). The estimated magnitude ranges between 7.2 and 7.7^12,13^.

We describe CI architecture (i.e. its distribution, thickness and vertical and lateral variations of lithofacies) and explore the role of eruptive and transport mechanisms as well as topography, weather, and surficial water as factors that influenced deposition from the pyroclastic current. Ignimbrites are considered to be emplaced by concentrated pyroclastic flows^19,20^ or dilute and turbulent pyroclastic currents^21–23^. Because of their complexity, the physics of these flows are still poorly understood. Experimental approaches^24–28^ and numerical formulations^29,30^ have been used to simulate transport and emplacement of this type of gravity current. Field and laboratory data allowed previous studies to conclude that the CI was deposited from a dilute pyroclastic current^31–33^. We concur with these authors on the dilute nature of the CI transport system and use their conclusions as a starting point. In this paper, we integrate a model^33^ addressing the turbulent and dilute nature of the CI pyroclastic current, during the emplacement of the CI ground layer, with new data (lithofacies and their grainsize characteristics) extending its application to the whole medial ignimbrite sequence. The study highlights how the CI ignimbrite sequence was assembled and the main internal and external factors that influenced its sedimentation, which can in turn help to constrain the general behaviour of large pyroclastic currents.

### Stratigraphy and regional distribution of eruptive products

The Campanian Ignimbrite eruption produced two main phases: a sustained eruption column, that emplaced a widespread Plinian Pumice Fall deposit east of Campi Flegrei^18,34,35^, followed by a collapsing column phase that distributed a massive, mostly welded, grey ignimbrite sheet in medial areas, 10 to 80 km from source^14–16,31–33,36,37^ and a proximal accumulation, within 10 km, of lithic breccias, welded horizons and unconsolidated pumiceous deposits along the Campi Flegrei caldera rim (Breccia Museo formation^38,39^, Fig. 1). Field and chemical data constrain the emplacement condition of the aggrading ignimbrite from a single sustained current (see below for discussion). A widespread tephra layer (Y5), extending from the eastern Mediterranean Sea to Russia, is associated with both the sustained (co-Plinian) and collapsing (co-ignimbrite) phases^40–42^.

The pyroclastic sequence of the Campanian Ignimbrite eruption has been subdivided into ten stratigraphic units, nine identified in the pyroclastic current deposits plus the basal pumice fall deposit^16^ (Fig. 2). The proximal deposits can be observed in limited exposures along the Campi Flegrei caldera rim (Fig. 1) directly overlying the pumice fall deposit. The entire sequence, up to 70 m thick, makes up the Breccia Museo formation (Fig. 2).

The medial pyroclastic current deposits are found in the Campanian Plain and on the Southern Apennine chain, up to ~ 80 km from the vent area, and consist of 4 distinctive units resting on the pumice fall deposit to the East of Campi Flegrei and on a paleosol or sedimentary rocks in the other sectors^16^ (Fig. 1). The medial ignimbrite sequence begins with an unconsolidated stratified ash flow (USAF) deposit containing fine pumice lapilli (Fig. 2). A fines-poor, lithic and crystal-rich bed occurs at its base^33^ (ground layer). USAF passes upwards into the main and most voluminous welded grey ignimbrite (WGI), a grey, welded tuff or lapilli tuff with an unconsolidated basal horizon. WGI contains both scoria and pumice clasts (vesicularity 40 to 68% and 72 to 82% respectively). WGI shows lateral and vertical variation of juvenile clast types (scoria or pumice) and welding degree. WGI passes upwards into the lithified yellow tuff (LYT), the product of post-depositional zeolitization of the upper, unconsolidated part of the ignimbrite^17,43^. This unit is composed of a massive, yellow, lithified tuff or lapilli tuff with rounded juvenile lapilli. It shows the same lateral variation of juvenile clast types as the underlying WGI. The uppermost unit, a coarse pumice flow deposit (CPF), is an unconsolidated, light grey, poorly sorted, pumice lapilli-and-blocks deposit. Locally, a reverse graded pumice concentration zone (pcz) can be observed at the top. Chemically, medial deposits, are trachytic in composition and show a general decreasing trend of evolution both with stratigraphic height and distance from the source^44,45^. Conversely, the upper coarse unit has a heterogeneous composition^45^.

Medial units have a quite different areal distributions and are dispersed in different sectors around Campi Flegrei (Fig. 1). USAF is ubiquitously present from proximal exposures as far as 70 km from source (including the ground layer at its base). WGI is also dispersed in all sectors from 10 to at least 80 km from source. Variation of juvenile clasts (scoria and pumice) in WGI, and in the lithified yellow tuff, appears to be strongly influenced by paleotopography at regional scale. Up to 50 km from source, on average, most of WGI thickness is characterized by dark grey to black, moderately vesiculated, rounded, locally reverse graded scoria lapilli and blocks. This “scoria-rich ignimbrite” is moderately to strongly welded and shows a lateral transition to a proximal intensely welded tuff, Piperno unit, along the eastern sector of Campi Flegrei caldera rim^46^ (Fig. 2). It is mainly distributed in the Campanian Plain and confined by the mountain ridges surrounding the plain. In this area, the lowermost part of WGI, from decimetres to a few meters, is composed of light grey, pumice lapilli embedded in the unconsolidated to sintered matrix (Fig. 2). Farther from the source and beyond the mountain ridges, > 50 km on average, the whole thickness of WGI is unconsolidated to incipiently welded and contains rounded, highly vesiculated, light grey, coarse to fine pumice lapilli. A boundary between the scoria-rich and pumice-rich WGI is shown in Fig. 1. This boundary is not a sharp physical boundary, but it has to be considered as a transition zone where a change in the type of juvenile clast has been observed. The boundary is stretched northward to the Roccamonfina volcano area where the current flowed across the Campanian Plain and flat valleys without the obstruction of high relief. Conversely, in a wide area from N-NE to SE of Campi Flegrei, the limit is strongly influenced by high mountain ridges (up to 1500 m a.s.l.). In this area, the limit is parallel to the ridges and is, locally, deflected by the main valleys transverse to the ridges. The lithified yellow tuff is distributed in a wide sector N-NE to SE and of up to 70 km from Campi Flegrei. The Coarse Pumice Flow is the less distributed medial unit; it is confined 30–37 km from source, including Capri Island.

## Results

### Campanian Ignimbrite architecture

**Figure 3:**
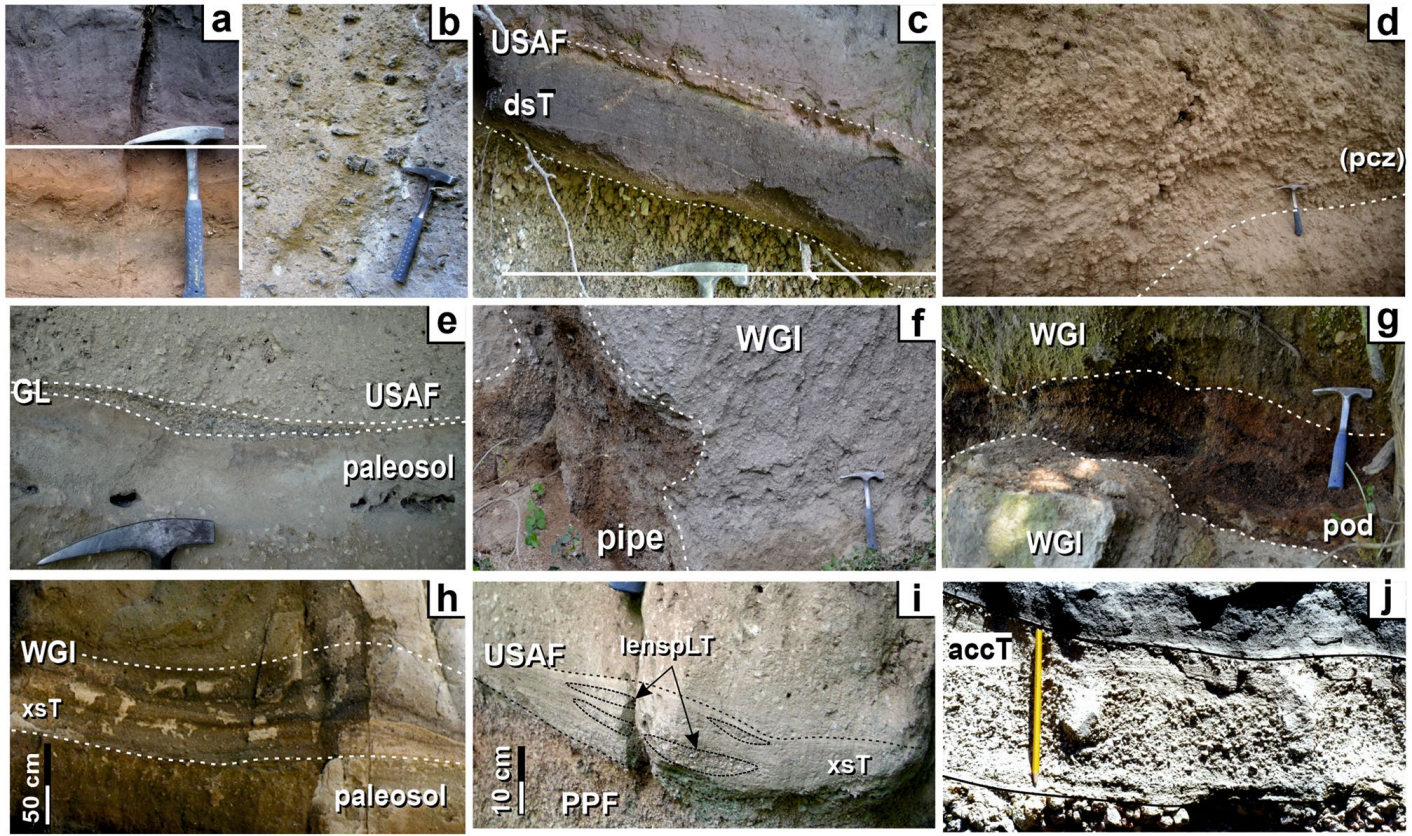
Lithofacies recognized in the medial pyroclastic current deposits of the Campania Ignimbrite eruption: (**a**) Massive tuff; (**b**) Massive lapilli tuff; (**c**) Diffuse-stratified tuff; (**d**) Pumice-rich lapilli tuff showing a pumice concentration (pcz) zone at the top; Fines-poor lithic lapilli tuff occurs as Ground Layer, GL (**e**), at the base of the ignimbrite sequence or pipes (**f**) and pods (**g**) intercalated in the main ignimbrite body (WGI unit); Cross-stratified tuff lithofacies is characterized by cross-stratification/lamination and bedforms with wavelength from 4 m (**h**) to few centimeters (**i**). Pockets of fines-poor, sub-angular, fine pumice lapilli may be present between the laminae of small bedforms forming the “Lenses of pumice lapilli” lithofacies (**i**); (**j**) Accretionary lapilli-bearing lithofacies. Figure is generated using CorelDRAW Graphics Suite 2020 for Windows v. 22.0.0.412 (https://www.coreldraw.com/it/product/coreldraw/).

**Figure 4:**
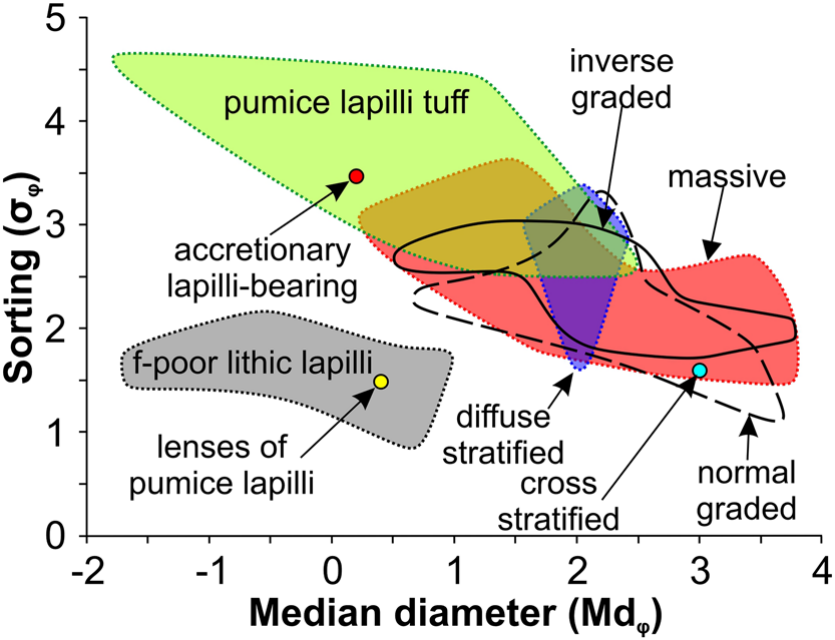
Median diameter (Md_φ_) vs sorting (σ_φ_) diagram showing the grainsize characteristics of lithofacies recognized in the medial pyroclastic current deposits of the Campanian Ignimbrite eruption. Figure is generated using CorelDRAW Graphics Suite 2020 for Windows v. 22.0.0.412 (https://www.coreldraw.com/it/product/coreldraw/).

CI medial ignimbrite sequence comprises seven lithofacies based upon the different sedimentary structures (Table 1 and Fig. 3). A comprehensive description and interpretation of each lithofacies is reported in Supplementary Material while grain-size characteristics are shown in Fig. 4. Each specific lithofacies may occur in different units. In the entire ignimbrite sequence, the most common lithofacies is a poorly sorted (1.6 < σ_φ_ < 3.6; Fig. 4) unconsolidated to sintered to welded, massive (lapilli) tuff (mT or mLT, Fig. 3a and 3b) locally showing gas-escape structures, various coarse tail grading patterns (Table 1) and grain fabrics from weakly developed to chaotic or entirely lacking. Diffuse-stratified tuff (dsT, Fig. 3c) is composed of sub-parallel, well to poorly sorted (1.6 < σ_φ_ < 2.3) laminae having sharp to diffuse upper and lower boundaries. This facies is typical in the basal stratified unit and rare in WGI. Pumice-rich lapilli tuff (pLT, Fig. 3d) is massive and poorly to very poorly sorted (2.6 < σ_φ_ < 4.6) deposits containing abundant, coarse-tail graded, pumice lapilli to blocks, locally forming a pumice concentration zone at the top, and very subordinate mm-sized, slightly normal graded, lithic lava clasts. Pumice blocks have variable chemical compositions^45^. This facies occurs only in the upper coarse unit. Fines-poor lithic lapilli tuff (f-poorlLT) comprises massive, well sorted (0.96 < σ_φ_ < 2.07), unconsolidated, lithic- and crystal- rich deposits occurring as ground layer (Fig. 3e) at the base of the sequence or as pipes (Fig. 3f) and pods (Fig. 3g) intercalated in the ignimbrite (WGI) along riverbeds.

**Table 1:** Summary description and interpretation of CI lithofacies.

Lithofacies	Description	Interpretation
Massive tuff (mT) or massive lapilli tuff (mLT) (isc/ip; np; il/nl)	*Structure and lithology:* massive, matrix-supported, unconsolidated to sintered to welded (or lithified) deposits containing fine (mT) to coarse (scoria or pumice) lapilli and blocks (mLT) and very subordinate lithic fragments (mainly lava and rare limestone) set in the matrix. Gas-escape structures and columnar jointing occur *Thickness:* few tens of centimeters to tens of meters *Grading:* ungraded (both juvenile and lithic clasts); inverse graded scoria (isc) or pumice (ip) associated with inverse (il) or normal (nl) graded or ungraded lithic; normal graded pumice (np) always associated with normal graded lithic *Sorting:* mainly poorly sorted (1.6 < σ_φ_ < 3.6) *Directional grain fabric:* weakly-developed to chaotic to absent, rarely moderately developed *Unit:* USAF, WGI and LYT	Aggradation from high concentrated, fluid-escape dominated flow-boundary zone in absence of directional grain fabric Intermediate conditions between fluid-escape and grain-flow dominated when a weakly to moderately developed directional grain fabric is present^21,27,47–51^ Gradation is related to waxing (inverse of both juvenile and lithic clasts) or waning (inverse juvenile clasts and normal lithic or both normal) current conditions^21^
Diffuse-stratified tuff (dsT)	*Structure and lithology:* stratified/laminated to diffuse-stratified/laminated, unconsolidated to compacted, deposits containing very fine pumice lapilli set in the matrix. Laminae are sub-parallel and mm- to sub-mm-thick *Thickness:* 0.2 to 1 m *Grading:* ungraded *Sorting:* well to poorly sorted (1.6 < σ_φ_ < 2.3) *Directional grain fabric:* weakly to moderately developed *Unit:* typical in USAF, rare in WGI	Intermediate conditions between traction- and grain-flow dominated flow-boundary zone^9,21,52^
Pumice-rich lapilli tuff (pLT)	*Structure and lithology:* massive, matrix-supported, unconsolidated deposits containing abundant rounded, highly to extremely vesiculated (vesicularity between 73 and 86%) pumice lapilli to blocks *Thickness:* up to 6 m *Grading:* inverse grading of pumice, locally, forming a pumice concentration zone (pcz) at the top; faint normal grading of lithics *Sorting:* poorly to very poorly sorted (2.6 < σ_φ_ < 4.6) *Directional grain fabric:* absent *Unit:* CPF	Fluid-driven, selective filtering (buoyancy) of pumice in a high concentrated flow-boundary zone. Deposition occurs from a waning current. A pcz results when this process is particularly efficient^21,49^
Fines-poor lithic lapilli tuff (f-poorlLT)	*Structure and lithology:* fines-poor, lithic and crystal-rich and pumice-poor, unconsolidated, massive deposits occurring at the base (ground layer) or intercalated (pipes and pods) in the ignimbrite sequence *Thickness:* 0.5 to 21 cm (ground layer); up to 1.2 m wide and 2.5 m high (pipe); up to 1.2 m thick and 8 m long (pod) *Grading:* ungraded *Sorting:* well to very well sorted (0.96 < σ_φ_ < 2.07) *Directional grain fabric:* absent; locally weakly developed in presence of elongated, fine to very fine lithic lapilli. (ground layer) *Unit:* at the base of USAF (ground layer) or intercalated in WGI along riverbeds (pipes and pods)	Segregation of dense and heavy fragments and elutriation of fine vitric material *Ground layer*: Segregation occurs at current front due to ingestion and thermal expansion of cold air^33,53,54^ *Pipes and pods*: Segregation occurs due to flashing of water to steam caused by interaction of superficial water (rivers and creeks) and the hot pyroclastic current^55,56^
Cross-stratified tuff (xsT)	*Structure and lithology:* cross-stratified/laminated, unconsolidated to welded, deposits containing fine juvenile clasts and forming large to small bedforms *Thickness:* few centimeters to < 1 m; large bedforms are up to 4 m in wavelength and 20 in amplitude; small bedforms are up to 30 cm in wavelength and 3 cm in amplitude *Grading:* ungraded *Sorting:* well sorted (σ_φ_ = 1.6) *Directional grain fabric:* moderately to well developed *Unit:* USAF; rare in WGI	Low-concentrated, traction-dominated flow-boundary zone in a highly unsteady current^9,21,52,57–59^. Welding may have occurred during the final stages of deposition (syn-depositional) of a formerly particulate flow^21,60,61^
Lenses of pumice lapilli tuff (lenspLT)	*Structure and lithology:* fines-depleted and clast-supported, unconsolidated, massive lenses composed of sub-angular, highly vesiculated, fine pumice lapilli occurring between the laminae of xsT facies *Thickness:* up to 3 cm thick and 25 cm long *Grading:* ungraded *Sorting:* well sorted (σ_φ_ = 1.5) *Directional grain fabric:* absent *Unit:* towards the base of USAF only where this unit rests on pumice fall deposit	Portion of the pumice fall deposit eroded by the turbulence and re-sedimented after a brief transport from a low concentration, traction-dominated flow-boundary zone^9,52^
Accretionary lapilli-bearing tuff (accT)	*Structure and lithology:* Unconsolidated, massive beds rich in accretionary lapilli having concentric layers. Accretionary lapilli are up to 15 mm in diameter and accumulate at the base of each bed and decrease upward *Thickness:* few decimeters *Grading:* ungraded *Sorting:* poorly sorted (σ_φ_ = 3.5) *Directional grain fabric:* absent *Unit:* WGI	Localized rainstorms affected the ash-rich transport system of the pyroclastic current^62^ causing ash aggregation around a fine pumice fragment acting as a core. Falling through the pyroclastic current, accretionary lapilli accreted by successive concentric layers^63^

Cross-stratified tuff (xsT) is composed of well sorted (σ_φ_ = 1.6) deposits forming large to small bedforms. We report for the first time a large bedform, up to 4 m in wavelength and 20 cm in amplitude (Fig. 3h), in the weakly welded grey ignimbrite (WGI), 50 km from the vent (sec. 17—Roccabascerana). The sandwave bedset occurs at the base of WGI and vanishes upwards. It nucleates at the top of a small, local ridge and grades laterally into a massive, 4 m thick, valley-ponding deposit with well-developed columnar jointing. The bedform comprises gently dipping lee-side layers overlain by layers preserved on both sides of the bedform so that single strata can be followed from the stoss to the lee side. The undulation has gently dipping sides with rounded and ‘stationary’ crests. Internally, this bedform consists of massive layers made of an unsorted mixture of ash and minor lapilli clasts. Smaller bedforms, up to 30 cm in wavelength and 3 cm in amplitude occur in the basal stratified unit (Fig. 3i). Fines-depleted, massive, well sorted (σ_φ_ = 1.5) lenses of sub-angular, highly vesiculated pumice lapilli (LenspLT, Fig. 3i) locally occur between the laminae of small bedforms in the lower part of the basal stratified unit, only where this unit rests on pumice fall deposit.

A rare facies is the accretionary lapilli-bearing (accT, Fig. 3j). This unconsolidated, poorly sorted (σ_φ_ = 3.5) facies is confined on the slope of mountains along a NW–SE transect ranging from 44 to 58 km from the vent. It consists of few decimeter thick ash beds with erosive contacts. Accretionary lapilli are accumulated at the base of these layers and decrease upwards. This facies grades laterally into a massive, valley-ponding welded deposit up to 8 m thick.

Several indicators define the flow dynamics of the parental current. The presence of: (a) multiple vertical facies; (b) deposits beyond a stretch of sea or 1500-m-high mountain ridges; (c) strongly erosive basal contact^16^; (d) strong ash elutriation with a calculated vitric loss of 65%^12^ are consistent with a dilute, turbulent, high velocity pyroclastic current^31–33^. In this current, particles are mainly transported in suspension by turbulence (transport system) and sedimenting progressively from a basal depositional system.

Our systematic facies description indicates that the depositional system shows spatial and temporal variability in many parameters (e.g. concentration, components) discussed below. Hereafter, we refer to flow-boundary zone as transitional zone that includes the lowermost part of the current and the uppermost part of the forming deposit, where lithofacies characteristics are determined^21^.

The main facies (mT or mLT) exhibits a variety of juvenile (scoria and pumice) clasts and lithic grading patterns. The occurrence of gas-escape structures and lack of directional fabrics, in most of the outcrops, is consistent with deposition from a fluid-escape dominated flow-boundary zone. The presence, locally, of a weakly-developed directional grain fabric marks the temporary transition to a granular flow-dominated flow-boundary zone^21,27,47–49^. Inverse grading of both juvenile and lithic clasts indicates the sedimentation from waxing current. Similarly, inverse grading of juvenile clasts and normal grading of lithics, or both normal graded, can be attributed to the same flow-boundary conditions in a waning current^21^. In both cases, settling of particles occurred through a high-concentrated flow-boundary zone. A very efficient selective filtering (density segregation) is responsible for the inverse grading of pumice (and normal grading of lithics) in the pLT facies up to form an upper pumice concentration zone. The buoyancy effects hindered the settling of very coarse, highly to extremely vesiculated juvenile clasts (vesicularity 80–86%) until the current completely wanes^21,49^. The compositional variability of the pumice blocks in the pumice concentration zone support this model^45^. Fines-poor, lithic- and crystal-rich deposits (f-poorlLT) are related to segregation of heavy components and elutriation of fine vitric material^53^. Segregation occurs at the current front^33,53,54^ (ground layer) or due to flashing of water to steam caused by interaction between the hot pyroclastic current and local superficial water^55,56^ (pipes and pods). It is important to stress that this lithic-rich facies is not related to any caldera collapse episode which occurred later in the course of the CI eruption, and that the abundance of heavy materials is therefore associated with depositional processes. A diffuse faint bedding (dsT) indicates intermediate conditions between granular flow-dominated and traction dominated- flow-boundary zone due to a variable suspended load fallout rate^64^, while cross-stratification and bedforms (xsT) indicate a transition to purely traction condition. Strongly erosive basal contact of each lamina in xsT facies are commonly associated with a pulsatory (unsteady) behaviour of a dilute pyroclastic current during the deposition from a low-concentration flow-boundary zone^9,21,52,57,65^. Individual laminae aggraded at the base of a dilute pyroclastic current when particle clusters, segregated from the homogeneous mixture, settled downward^58,59^. The bedform observed in the welded WGI is an evidence that agglutination process is compatible with traction. The welding may have occurred during the final stages of deposition of a formerly particulate flow^21,60^. The occurrence of lenses of sub-angular, highly vesiculated pumice lapilli (lenspLT) between the laminae of the xsT, only where the basal stratified unit rests on the pumice fall deposit, indicates that such lenses may be portions of underlying deposit eroded by the turbulence and re-sedimented after a brief transport in a low concentration, traction-dominated flow-boundary zone^52^.

The localized distribution of accT facies that passes laterally to a sintered, valley-ponding deposit indicates that it is not the product of a phreatomagmatic phase at the vent. The distribution in a well-defined topographic setting, parallel to the Apennine ridge, suggests that localized rainstorms may have affected the ash-rich pyroclastic current dropping the pyroclastic current temperature and promoting ash aggregation^62^. The unconsolidated occurrence of this facies indicates that the emplacement temperature was below the zeolitization threshold (120 °C), preventing the development of the lithification process^17^. Ash aggregated around a fine pumice fragments, acting as a core, forming the embryo of the accretionary lapilli. Accretionary lapilli fell through the pyroclastic current transport system and accreted by successive concentric layers^63^.

**Figure 5:**
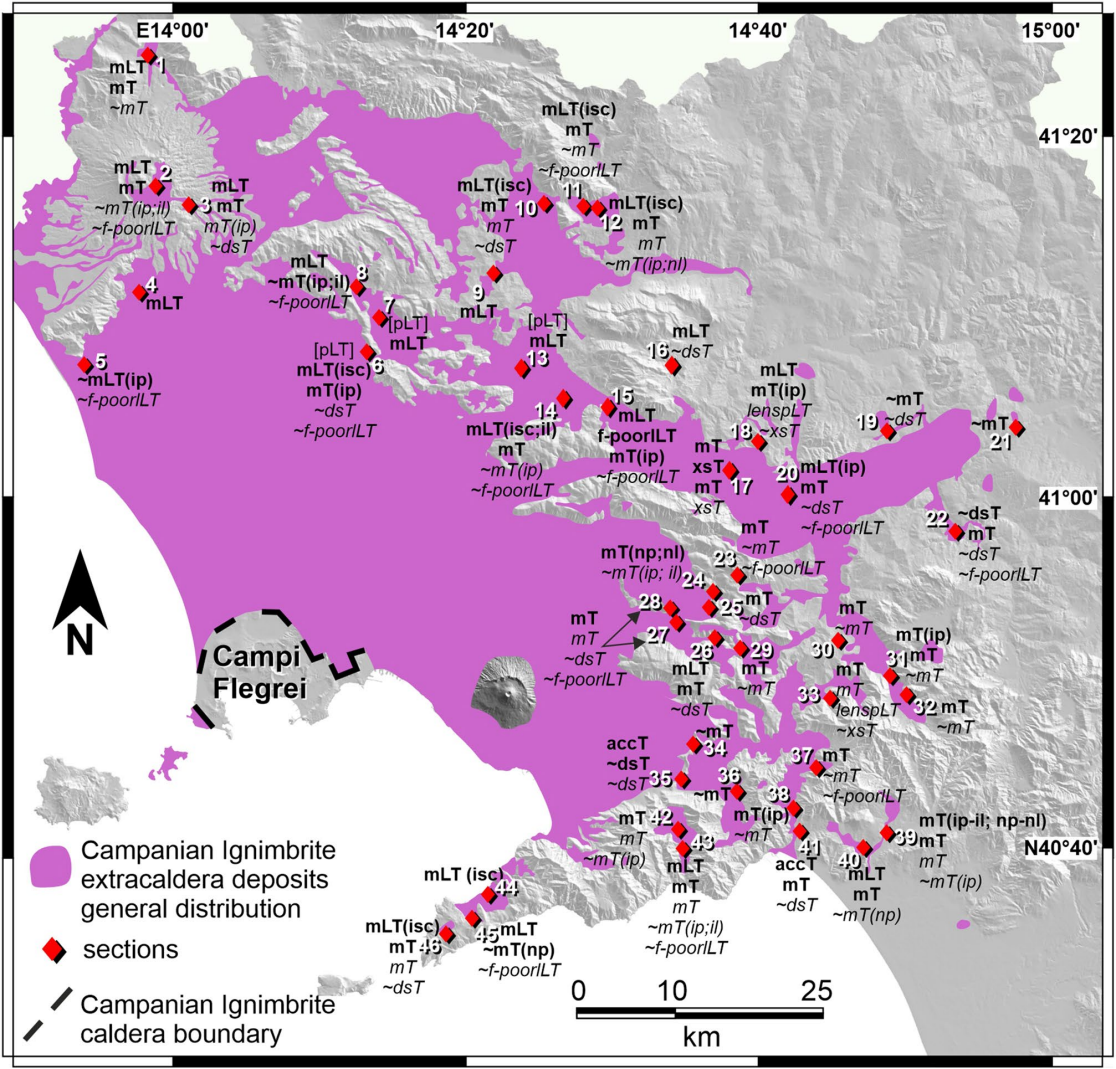
Distribution of lithofacies recognized in the medial pyroclastic current deposit of the Campanian Ignimbrite eruption throughout the investigated area and in each studied section. Section numbers and location as in Fig. 1. Two or more superimposed lithofacies at same location represent a vertical facies variation. Italic refers to facies recognized in the unconsolidated stratified ash flow deposit, bold refers to WGI while facies recognized in coarse pumice flow deposit are reported in square brackets (sec. 6, 7 and 13). Lithified yellow tuff facies are not reported because are the same of the underlying WGI. Facies abbreviation: f-poorlLT (fines-poor lithic lapilli tuff); mT or mLT (massive tuff or massive lapilli tuff), isc/ip (inverse graded scoria/pumice), np (normal graded pumice), il/nl (inverse/normal graded lithic); dsT (diffuse stratified tuff); xsT (cross stratified tuff), pLT (pumice lapilli tuff); lenspLT (lenses of pumice lapilli tuff); accT (accretionary lapilli-bearing). Figure is generated using CorelDRAW Graphics Suite 2020 for Windows v. 22.0.0.412 (https://www.coreldraw.com/it/product/coreldraw/).

A map reporting the distribution of lithofacies in the investigated area and the vertical facies variation in each studied section is shown in Fig. 5. Distribution of lithofacies shows a remarkable lateral persistence in most of the units at regional scale (Fig. 5) consistent with an overall uniform behaviour of the pyroclastic current. The presence, along the edges of the WGI distribution, of stratified and diffuse stratified facies, in contrast with massive facies observed closer to the source (Fig. 5), marks the lateral variation from a fluid-escape dominated to a low-concentrated, traction-dominated flow-boundary zone. This spatial transition indicates that the pyroclastic current diluted with the distance. We have observed four lateral facies association at the outcrop scale: (a) sandwave to diffuse stratified to massive; (b) diffuse stratified to inverse graded; (c) massive to inverse graded; (d) stratified to accretionary lapilli bearing to massive tuff. Local downcurrent changes are related to increase (c) or decrease (a, b, and d) in flow velocity caused respectively by an increase or decrease in slope steepness or topographic barrier^21^.

Vertical variations (three or more superimposed facies) can occur in few centimeters or meters in a single outcrop. Three main vertical facies associations have been identified throughout the ignimbrite sequence: (a) fines-poor lithic lapilli to diffuse stratified or massive or inverse graded; (b) cross-stratified or diffuse stratified to massive or normal- to inverse-graded; (c) massive to inverse graded. These vertical variations define a dominant trend during which the particle flux into the flow-boundary zone was progressively increasing, at a fixed point, and records the rises of concentration and the increasing importance of fluid-escape condition with time. This is supported by a vertical change from deposits with well-developed fabric at the base, to deposits without fabric higher up. An increase in the rate of supply^64,66^, from the upper dilute and turbulent transport system towards the base of the current, is responsible for the suppression of any turbulence in the flow-boundary zone. In the overloaded flow-boundary zone, the increase of ratio between the sedimentation rate and transportation rate^27^ may have played an important role.

### Nature and dynamics of the CI pyroclastic current

The absence of any fall layer within the ignimbrite, unlike Novarupta ignimbrite and Bishop Tuff, which are both characterized by numerous fall layers within the ignimbrite^5,7^, indicates that no resumption of the Plinian activity occurred during the eruption after the emplacement of the basal pumice fall. Throughout the medial sequence, no co-ignimbrite ash layers or sub-aerial erosive surfaces, indicating an even brief pause in the passage of the current, have been found. These evidences together with the vertical and lateral transition from more evolved to less evolved products^45^ suggest a mechanism of progressive aggradation from a single current.

**Figure 6:**
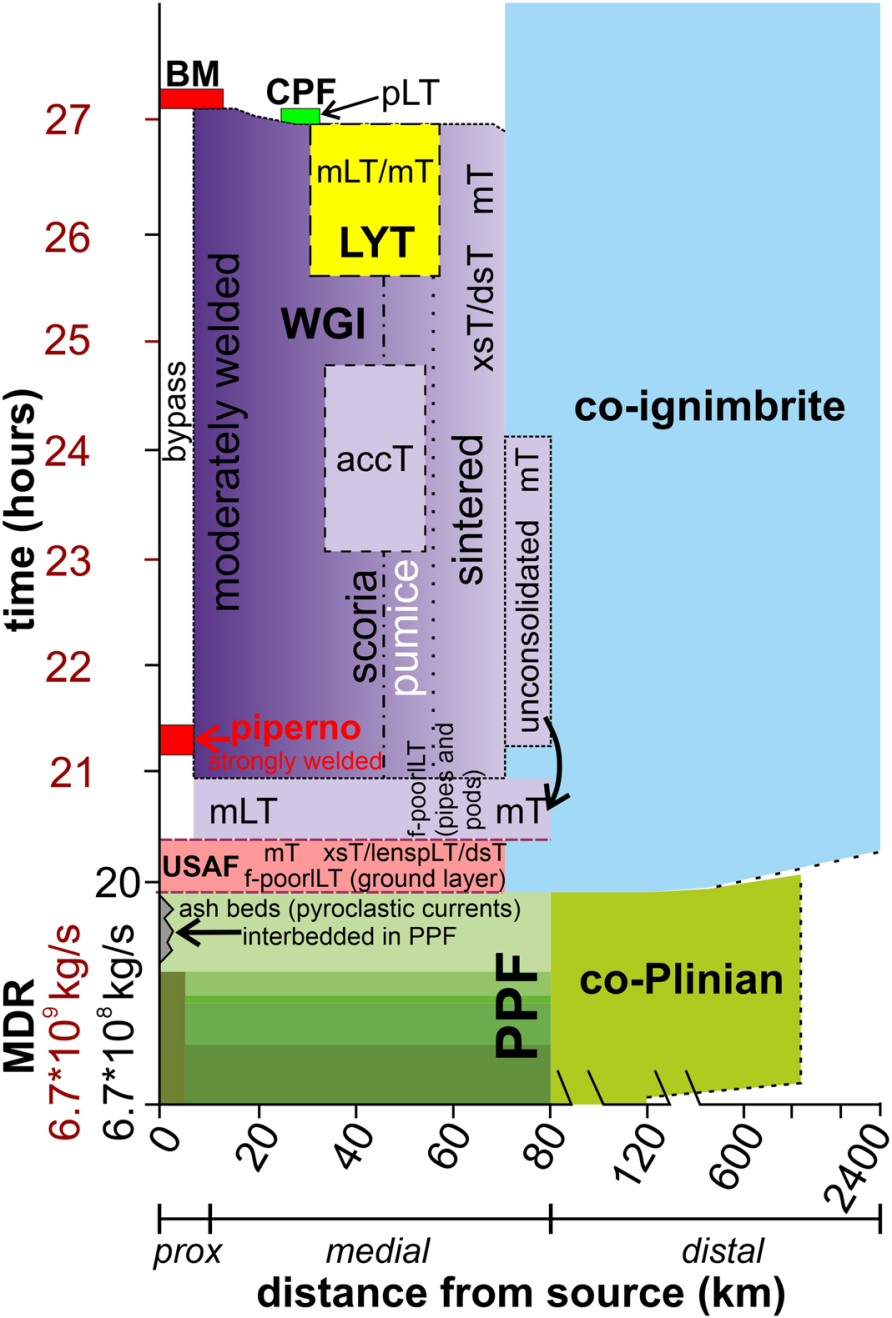
Time-distance plot showing lithofacies architecture of the Campanian Ignimbrite. Pyroclastic current units are deposited during waxing and waning phases. Emplacement time, at increasing distance from the vent, is defined using a cloud spreading velocity of 130 m/s^67^, and a 220 m/s pyroclastic current velocity for the CI current^23^. The values of mass discharge rate refer to the Plinian phase (black)^18^ and ignimbritic phase (red)^67^, respectively. Note that due to the time scale chosen and the high velocity of spreading of the products of both phases, the boundaries between the different units are almost horizontal. Only for the distal co-Plinian and co-ignimbrite ashes it is possible to appreciate a diachronic sedimentation. Figure is generated using CorelDRAW Graphics Suite 2020 for Windows v. 22.0.0.412 (https://www.coreldraw.com/it/product/coreldraw/).

A > 10^9^ kg/s mass discharge rate withdrew 64 km^3^ of trachytic magma, erupted during the collapsing phase, in 7 h^45,67^. A time-distance plot (Fig. 6) synthesizes the emplacement of all units associated to the Campanian Ignimbrite eruption and the lateral and vertical facies variations of the CI pyroclastic current medial deposits. A > 1.5 km deep, turbulent and dilute pyroclastic current^33^ spread radially over the whole rugged region. The CI current had a high erosive capacity as demonstrated by the erosion rate of the underlying pumice fall deposit^16^. A fines-depleted, lithic- and crystal-rich layer (ground layer) was emplaced radially around the source. It exhibits nearly circular isopleths^33^. Traction conditions of the flow-boundary zone produced a stratified to diffuse stratified, unconsolidated, deposit above the ground layer. During this phase, the eruptive mixture contained a well vesiculated juvenile (pumice) component and the current waxed and prograded radially (at least 70 km from source, inset of Fig. 1). Further propagation of the pyroclastic current and increase of the inundated area, possibly caused by an increase in eruptive flux, occurred during the emplacement of the main ignimbrite units (WGI and overlying Lithified Yellow Tuff). During the climax, the current had a maximum runout of at least 80 km (WGI; Fig. 1) from Campi Flegrei. A transition between traction-dominated and granular flow- or fluid-escape-dominated flow-boundary zone influenced the emplacement of the main phase of the CI eruption.

By comparing some features such as thickness, juvenile clasts type, and range of vertical chemical composition of WGI and its proximal, high-grade counterpart, Piperno, an important aspect of transport/depositional behaviour in more proximal areas arises. Piperno is much thinner than WGI (few metres vs tens of metres, Fig. 2), contains scoriaceous fragments throughout its thickness, instead of a pumice-rich base as in WGI, as well as a narrower vertical range (581–758 ppm) in zirconium (Zr) than WGI (271–719 ppm)^39,45^. All these features indicate that Piperno does not represent the proximal equivalent of the whole WGI unit but was emplaced in a short interval during the beginning of sedimentation of the more evolved, scoriaceous part of WGI. Therefore, during most of the WGI eruptive phase, the parental pyroclastic current produced a bypass zone (Fig. 6; area where the pyroclastic current passes without depositing) around the Campi Flegrei caldera while massive, locally graded, moderately to strongly welded, thick successions accumulated in medial areas.

Segregation processes of juvenile clasts types (scoria and pumice) due to different density (and vesicularity) and the effect of paleotopography at regional scale were responsible for the lateral transition from scoria-rich to pumice-rich WGI. Scoria clasts were segregated towards the lower part of the dilute pyroclastic current transport system. The scoria-rich lower part of the pyroclastic current was mainly blocked by the mountain ridges^68^ bounding the southern-eastern sector of Campanian Plain and partly channelized into the valleys transverse to the main ridges. In the northern sector of the Campanian Plain (towards Roccamonfina volcano, Fig. 1), the scoria-rich base of the CI current was able to reach greater distances due to the absence of high topographic barriers. Light pumice clasts were fully supported in the upper part of the pyroclastic current transport system, overpassed the ridges and were emplaced in the outermost areas. The pumice block-rich pyroclastic current front retrograded further sourcewards leaving an incoherent and coarse deposit above the welded/lithified facies between 30 and 37 km N-NE of Campi Flegrei and possibly on the island of Capri (Fig. 1). The final waning phase, during the later stage of the eruption, accumulated a lithic-rich, proximal sequence (Breccia Museo) around the collapsing Campi Flegrei caldera rim (Fig. 1). The unwelded top of the ignimbrite was later lithified due to post-emplacement processes forming a yellow zeolitized facies in a sector N-NE to SE of, and up to 70 km from, Campi Flegrei.

The pyroclastic current underwent time-related temperature variations. Hereafter, we refer to the glass transition temperature^69^ as minimum threshold of pyroclastic current temperature for welding to occur. In the early stage, well vesiculated juvenile clasts were discharged and emplaced below the glass transition temperature (unconsolidated stratified basal unit to sintered WGI base). The vertical transition from unconsolidated to welded deposits was related to an increase in temperature possibly due to the rise of the percentage of collapsed mass during the eruption^70^. Some authors^36^ consider the load compaction^61^ as the primary cause of the variable, vertical welding degree of the grey ignimbrite. The occurrence of thin (less than 1 m) welded deposits on top of a local ridge is not compatible to load compaction but rather to syn-depositional agglutination processes of pyroclasts at the glass transition temperature. Hot and sticky particles coalesced along contact points just before final deposition. The shear stress exerted by the overriding turbulent current contributed to the efficiency of this process^21,71^. Decrease of welding of WGI even with the distance from the source testifies to a progressive space-related cooling of the current. Subsequently, the pyroclastic current temperature progressively dropped, producing an unconsolidated ash and lapilli deposit on top of the welded ignimbrite, later lithified. The zeolitization process was caused by percolation of meteoric water in a deposit having temperature between 120 and 230 °C^17,43^. In areas where WGI is not overlaid by the lithified facies we can assume that this unit was not formed because: (a) the emplacement temperature was high enough to prevent the formation of an unconsolidated deposit at top or (b) the unconsolidated upper part was eroded preserving only the welded part.

Chemically homogeneous, variably vesiculated juvenile clasts (scoria and pumice) were emitted simultaneously in the course of WGI emplacement^45^ suggesting that the different texture is not related to extraction of different magma batches but to variable magma rise velocity during the ascent along a cross section of a volcanic conduit in turn promoting different degassing^72^.

There are three main external factors affecting the Campanian Ignimbrite pyroclastic current behaviour.

#### Topography

High ridges (> 1000 m) at 45–50 km from the source represent a divide that separates WGI scoria-rich from pumice-rich deposits (Fig. 1). High topography influenced also sedimentological parameters with coarser and poorer sorted deposits (e.g. 0.3 < Md_φ_ < 2.8 and 1.8 < σ_φ_ < 3.6 in WGI) on slopes oriented towards the source relative to deposits on slopes opposite the vent (e.g. 1.6 < Md_φ_ < 3.4 and 1.3 < σ_φ_ < 3.2). These fractionations for density (scoria vs pumice), grain-size (coarse vs fine) and sorting (very poorly vs poorly) result from the dynamics of a stratified pyroclastic current, where coarse and dense clasts had higher concentrations toward the base of the current, and the presence of high ridges that exerted a blocking on the lower part of the current^68^.

#### Surficial water

Large pipes and pods within WGI, rich in lithics and crystals, are confined in riverbeds along valleys. They were generated by flashing of surface water to steam where the pyroclastic current crossed a river.

#### Rain

The localized distribution on the hillside of high reliefs of the accretionary lapilli-bearing facies passing laterally to a sintered, valley-ponding deposit excludes that it may be the product of a phreatomagmatic phase. The distribution, parallel to the Apennine ridge, indicates that rainstorms incorporated water in the pyroclastic current, cooling the current and promoting ash aggregation. This process was efficient but extremely localized, generating an overbank unconsolidated facies, rich in accretionary lapilli, while in few tens of metres, the valley-ponding facies shows its common sintered nature.

### General pyroclastic current behaviour

Insight into original flow dynamics can be obtained from facies association studies^73^. Facies associations and large-scale depositional architecture (CI example is summarized in Fig. 5), may help to understand how large pyroclastic currents evolve and may aid validation of numerical modeling.

The documented character of the CI lithofacies architecture (Fig. 5) can be used to constrain how a large pyroclastic current can evolve in time and space. Below, we summarize the key implications: (a) a pyroclastic current dilutes and cools with distance; (b) initial high velocity/capacity produces bypass areas around the source; (c) locations of deposition shift gradually (progradation and retrogradation) with time; (d) variations in welding degree are not related to the deposit thickness; (e) maximum erosion is related to the current front; (f) lateral variation of juvenile clasts, having different density (pumice and scoria), is related to filtering processes (buoyancy or sinking) acting during the transport and, eventually, contribution of topographic obstacles exerting a barrier effect; (g) superficial water or rain may influence the pyroclastic current transport and depositional system. Two corollaries of this integrated model imply that: (1) because of progradation, retrogradation and bypassing there is not a type sequence of all erupted products^74^; (2) vertical sampling in a ‘complete’ section comprising all stratigraphic units does not represent the whole compositional range of the erupted products.

## Methods

A total of 46 sections, located between 30 and 80 km from Campi Flegrei (Fig. 1), has been studied in detail throughout the Campania region as far as Roccamonfina volcano to North, Sele Plain to South and beyond the mountain ridges (Apennine Chain) bordering the Campanian Plain to East. Our fieldwork was mainly focused on CI outcrops in which the base of the ignimbrite is exposed allowing to study the whole sequence (the base in exposed in 42 out of 46 sections) and describe in detail the occurrence of different lithofacies and their vertical and lateral variations. For this reason, sections are located close to or beyond the reliefs bordering the Campanian Plain where the ignimbrite sequence thins. No sections have been studied in the Campanian Plain because CI is largely buried by younger volcanic deposits and/or alluvial deposits^75^. During this study, extensive sampling (153 samples) was carried out. Samples were collected in the unconsolidated to weakly sintered portions of all CI units to characterize each lithofacies even from a sedimentological point of view. Samples were dry-sieved down to 4φ (0.063 mm), at 1φ interval, while the hydrometer was used to discriminate particles down to 10φ (10^–3^ mm). A detailed and comprehensive description of the sedimentological features of all stratigraphic units is out of the scope of this paper. Here we report grain-size data useful for quantitatively describing the sedimentological features shown in the text. Deposits nomenclature follows^76^. A lithofacies may be preserved in an unconsolidated or lithified state and is designed with its lithified term (e.g. tuff).

## Supplementary information

Supplementary Information
