# Supplementary material for: Dynamics of large pyroclastic currents inferred by the internal architecture of the Campanian Ignimbrite
Source: Sci Rep. 2020 Dec 17;10:22230. doi: 10.1038/s41598-020-79164-7 (PMC7747645; doi:10.1038/s41598-020-79164-7)
Supplement: Supplementary file 1 — Supplementary Information [file 41598_2020_79164_MOESM1_ESM.doc]

**SUPPLEMENTARY INFORMATION**

**Dynamics of large pyroclastic currents inferred by the internal architecture of the Campanian Ignimbrite**

Claudio Scarpati^1*^, Domenico Sparice^1^, Annamaria Perrotta^1^

^1^Department of Earth, Environmental and Resources Sciences, University of Napoli Federico II, 80126 Napoli, Italy

^*^corresponding author, email: claudio.scarpati@unina.it

**Lithofacies and their interpretations**

Massive (Lapilli) Tuff (mT or mLT)

This lithofacies consists of massive, matrix-supported, loose to sintered to strongly welded, mainly poorly sorted (1.6< σ_φ_ <3.6) deposits occurring in beds ranging from few tens of centimeters to several meters. It is found mainly in WGI. Rounded, moderately (scoria fragments have 40 to 68% vesicularity) to highly (pumice fragments have 72 to 82 % vesicularity) vesiculated juvenile clasts, ranging from very fine (massive tuff, mT, ) to coarse lapilli (massive lapilli tuff, mLT, ) and finer lithic clasts showing different grading patterns are dispersed within the matrix. Vapour-phase structures and gas-escape structures were recognized. Lithic clasts, almost completely composed of fresh and altered lavas, are subordinate. This lithofacies is typical in valley­ ponding settings, filling valleys and topographic depressions and exhibits a chaotic to weakly-developed directional grain fabric. In many localities, both juvenile and lithic lapilli clasts, show different grading patterns. Grading patterns have been highlighted mainly by grain-size analyses although, locally, can be recognized directly in the field. *Inverse grading* of coarse tail is the most common pattern for juvenile (both scoria and pumice) clasts. A trend observed also in the whole sample (e.g. sec. 38 - Cologna, median diameter (Md_φ_) varies upward from 3.6 to 2.4φ). Thickness of layers varies from 25 cm to several meters, up to tens of meters. Inverse­ grading of juvenile clasts consists of fine-medium lapilli in the lower part and coarse lapilli to blocks in the upper part where clasts are, locally, up to 20 cm in diameter. Inverse ­grading of pumice (ip) or scoria (isc) clasts is associated with three types of grading patterns of lithic clasts: 1) inverse ­graded (il), 2) normal graded (nl) or 3) ungraded. *Normal grading* (e.g. sec. 45 - Cesarano, Md_φ_ varies upward from 1.3 to 2.3φ) is a much less represented pattern consisting of coarse-medium pumice lapilli in the lower part and fine lapilli in the upper part (np). Normal­ grading of juvenile clasts is always associated to a normal­ grading of lithic clasts.

*Interpretation*

The poor sorting and the absence of (or weak to moderate) directional grain fabric is consistent with the deposition from a high concentrated flow-boundary zone in which the traction is completely suppressed and the shear stress is weak or absent. These features associated to gas-escape structures indicate a fluid-escape dominated boundary layer as main emplacement mechanism^1,2^. Locally a vertical transition to a granular-flow dominated boundary layer occurred as testified by the presence of a weak to moderately developed directional grain fabric. The intensity of fabric strength marks the transition between the two flow-boundary conditions and indicates which condition was acting at any instant during the deposition^3^. Different grading patterns of light and heavy components and the absence of a directional grain fabric in most of the studied sites reflect an emplacement from a fluid-escape flow-boundary zone in a PC experiencing transition from waxing to waning conditions^3^. Inverse grading of both juvenile and lithic clasts indicates the sedimentation from waxing current. Similarly, inverse-grading of juvenile clasts and normal-grading of lithic clasts can be attributed to the same flow-boundary conditions in a waning current. In both cases, settling of particles occurred from a high-concentrated boundary layer. Normal-grading of juvenile and lithic clasts indicates a deposition from a highly concentrated basal part of a waning current. The presence of two juvenile components (pumice and scoria) is indicative both of a time-related compositional variation of the feeder magma and dynamics in the eruptive conduit^4^. Vertical and lateral change in juvenile clast types show a trend of decreasing degree of chemical evolution with increasing distance from the source and stratigraphic height suggesting a mechanism of progressive aggradation of a waxing current. Type and distribution of juvenile clasts throughout the ignimbrite sequence is further illustrate in the discussion section.

Diffuse-stratified tuff (dsT)

This lithofacies comprises well to poorly sorted (1.6< σ_φ_ <2.3), unconsolidated deposits, 0.2 to 1 m thick, showing a diffuse faint stratification/lamination. When resting on PPF, this facies appears to be strongly erosive. The lamination is defined by an alternation of thin sub-parallel, millimetre to sub-millimetre thick laminae composed of fine and coarse ash. The upper and lower surfaces of the laminae are locally sharp although they rapidly became diffuse when traced laterally. Laminae can contain very fine pumice lapilli having, in some cases, a faint common orientation. This facies is typical of USAF at the bottom of valleys or at the base of slopes and rare in WGI (e.g. sec. 22 - S. Mango sul Calore).

*Interpretation*

Sharp and erosive contact of each lamina indicate the deposition from a low-concentration, turbulent, traction-dominated flow-boundary zone. The rapid transition to a diffuse-stratification indicate that concentration in the flow-boundary zone increases due to a higher suspended load fallout rate^5^, generating a transition (intermediate conditions) between traction-dominated and granular flow-dominated boundary layer^3,6,7^ locally generating a faint directional grain fabric of fine pumice lapilli. Several rapid transitions from sharp to diffuse lamination are related to rapid and local variations of suspended load fallout rate in an unsteady current.

Pumice-rich lapilli Tuff (pLT)

This lithofacies comprises massive, matrix supported, poorly to very poorly sorted (2.6< σ_φ_ <4.6), unconsolidated deposits, up to 6 m thick, containing very abundant, highly vesiculated (vesicularity between 86 and 73%), rounded to sub-rounded pumice lapilli and blocks showing heterogeneous composition^4^. Locally, pumice clasts increase and became even coarser upward to form a clast-supported pumice concentration zone (pcz ) at the top of this facies (sec. 13 - Dugenta). A normal grading of much finer lithic lava clasts is associated to the striking inverse grading of pumice clasts (Md_φ_ varies upward from 2.1 to 1.2 to -1.7φ). Lapilli to block-sized, rounded, light grey lithic tuff fragments having a measured mean density of 0.88 g/cm^3^ are mainly present in the upper part of this facies. This facies has been recognized only in CPF unit.

*Interpretation*

The characteristics of this lithofacies together to its areal distribution close to the source area are interpreted as the products of a high concentrated boundary layer in which fluid-driven selective filtering resulted in buoyancy of coarse, highly vesiculated pumice clasts in a waning current. Buoyancy of large pumice clasts, due to a density contrast with the gas-ash mixture, produced a (inverse) size segregation of juvenile clasts while sinking of dense fragments produced a normal grading of finer lithic lava clasts. A very efficient selective filtering is also responsible for the pumice concentration zone locally found at the top of this facies. The buoyancy effect hindered the settling of very coarse and extremely vesiculated juvenile clasts until the current completely wanes^3,8^. The compositional variability of the pumice blocks in the pumice concentration zone support this model^4^. The occurrence of large lithic tuff fragments associated to pumice blocks in the upper part of this facies is attributable to their low mean density giving them a similar fluid-dynamic behaviour.

Fines-poor lithic lapilli tuff (f-poor lLT)

This lithofacies comprises fines-depleted, well to very well sorted (0.96< σ_φ_ <2.07), unconsolidated, lithic- and crystal-rich deposits occurring at different stratigraphic height in the ignimbrite sequence.

This facies is found at the base of the ignimbrite sequence as thin, massive layer, showing lower and upper erosional contact (ground layer, GL^9^). GL occurs, from more proximal locations to as far as 70 km from source, either as a thin laterally traceable layer (although its thickness may vary very rapidly) at the base of USAF or as little pockets preserved in depressions of the substrate. Thickness varies from 21 cm along the SW caldera rim to 0.5 cm in its more distal occurrence (sec. 22 - S. Mango sul Calore). GL is found in different topographic settings as at the base of slopes (sec. 5 - Mondragone), on top of high mountain ridges (e.g. sec. 23 - Acqua Fidia) and valley bottoms (e.g. sec. 14 - S. Agata dei Goti). GL shows a decrease of Md_φ_, maximum lithic (ML) and abundance of lithic material as well as an improvement of sorting coefficient with increasing distance from the source and altitude (see elsewhere^9^ for a detailed discussion of GL sedimentological characteristics). Average GL lithic and crystal abundance is 57 and 25 wt%. GL enrichment factor (calculated according to^10^) is > 30, almost five times greater than the associated ignimbrite (6.5^11^).

Locally, along riverbeds, this facies is found within the ignimbrite sequence (WGI) as pipes and pods . Pipes are commonly few millimetres wide and up to 1 m high although at Moiano (sec. 15) a pipe 1.2 m wide and up to 2.5 m high was observed. In the same location, several pods are intercalated in WGI. Pods are up to 8 m long and 1.2 m thick. Lithic lava clasts show a reddish-orange patina. Average abundance of lithic and crystal fragments in pipes and pods is, respectively, 47 and 34 wt%.

*Interpretation*

The GL at the base of the CI sequence is interpreted to have been formed by flow-front processes^12^ in a dilute PDC. The ingestion, heating and thermal expansion of cold air at the flow front result in a vigorous turbulence that facilitates the segregation of the heavy fragments (lithics and crystals) and elutriation of fine vitric material (responsible of the high enrichment factor). The progressive decrease in Md_φ_ and ML corresponds with lateral and vertical grading patterns of lithics within the current. The decrease of ML with increasing distance from the source was due to the loss in carrying capacity of the current travelling toward its distal end while the decrease of lithic and crystal content was due to the continuous sedimentation of the heavy components. The coarser clasts moved toward the basal part of the current, depositing in more proximal areas and at low altitude (250–300 a.s.l.), while the finer particles were fully supported by the turbulence in the upper part of the current, which was capable of surmounting high barriers. For a complete discussion about CI GL sedimentation process and current dynamics see^9^. Pipes and pods are interpreted to be generated by flashing of surface water to steam caused by the interaction between the hot PDC and local riverbeds^13^. This process locally caused a more efficient elutriation of fine vitric material forming degassing structures in the main CI unit (WGI) and was also responsible for the alteration of lithic (reddened) surface.

Cross-stratified Tuff (xsT)

We report, for the first time, the presence of well-developed, large bedforms in WGI unit of the Campanian Ignimbrite. They occur along the Roccabascerana-Tufara road, at almost 50 km from the vent at the top of a paleoslope. Here WGI is 1.5 m thick and weakly welded. It rests, with an erosive contact, on a stratified USAF. The sandwave bedset occurs at the base of WGI and vanish upwards. It is up to 4 m in wavelength and with an amplitude of about 20 cm. The undulation has gently dipping sides with rounded and ‘stationary’ crests. Internally, this bedform consists of massive layers made of an unsorted mixture of ash and minor lapilli clasts. The bedform comprises gently dipping lee-side layers overlain by layers preserved on both sides of the bedform so that single strata can be followed from the stoss to the lee side. It nucleates at the top of a small, local ridge and grades laterally into a massive, 4 m thick, valley-ponding deposit with well-developed columnar jointing. Smaller bedforms have been observed also in USAF. These sandwaves range from 22 to 30 cm in wavelength and 2 to 3 cm in amplitude and pass laterally to planar laminae, both having always an erosive basal contact. The internal stratification pattern is defined by asymmetric laminae, thicker and steeper up-flow with stoss side dipping at an angle up to 30° and gently dipping lee-side (< 10°). One sample collected in this facies at Montoro Superiore (sec. 33) is well sorted (σ_φ_ = 1.6).

*Interpretation*

Cross-stratification and erosive basal contact of each lamina are commonly associated to a pulsatory (unsteady) behaviour of a dilute PDC during the deposition from a low-concentration traction-dominated boundary layer^6,14,15^. Some authors^16,17^ suggested that, in some cases, the supply rate from the suspension may be more important than tractional transport in the flow-boundary zone causing higher aggrading rate on the stoss side than lee side of a bedform. Higher stoss-aggrading rate is favoured by particle trajectories, a combination of vertical fall and lateral component, in which stoss side is more exposed than lee side. The presence of fine-depleted lenses of fine pumice lapilli between the laminae, eroded from the underlying PPF (see lenspLT lithofacies), suggest a strongly erosive behaviour compatible with purely traction processes in a fully dilute boundary layer. The bedform observed in the welded WGI is an evidence that agglutination process is compatible with traction. The welding may have occurred during the final stages of deposition of a formerly particulate flow^3,18^. Alternatively, some of the bedforms observed in this study can be produced by dense granular flows; as recently simulated in laboratory experiments^19^.

Lenses of pumice lapilli (LenspLT)

This facies is composed of fines­-depleted, well sorted (σ_φ_ = 1.5), massive lenses of sub-angular, fine pumice lapilli (LenspLT, ). Locally (sec. 33 - Montoro Superiore, sec. 18 - Tufara) this facies occurs between the laminae of the cross-stratified lithofacies. The upper contact is strongly erosive. Such lenses, up to 25 cm long and 3 cm thick, occur at the base of USAF at the contact with the underlying PPF.

*Interpretation*

The angularity of the pumice clasts and the occurrence only towards the base of USAF, immediately above PPF, indicate that such lenses may be portions of PPF, on which the current was flowing, eroded by the turbulence and re-sedimented after a brief transport^6,14^. Such lenses are strictly associate to the bedform characterizing the xsT facies so we interpret them as emplaced from a dilute and turbulent, traction dominated boundary layer in a highly unsteady current.

Accretionary lapilli bearing (accT)

Locally, in an area between Nocera and Salerno, WGI is stratified and unwelded and rests on USAF. The thickness ranges between 2 m (Nocera) and 1 m (Salerno). WGI is characterized by few decimeters thick ashy beds with erosive contacts. Beds are poorly sorted with juvenile clasts represented by vesiculated pumice fragments. In both locations, the upper layer is very rich in accretionary lapilli. They accumulated at the base of these layers and decrease upwards. Accretionary lapilli are up to 15 mm in diameter and possess concentric layers of ash, often accumulated around a pumice clast (armoured lapilli), producing a smooth sub-spherical shape. This facies grades laterally into a massive, valley-ponding welded deposit up to 8 m thick. One sample collected in this facies is poorly sorted (σ_φ_ = 3.5). Accretionary lapilli have been already reported by Ort et al. (2003) at Altavilla Irpina, 55 km NE of Campi Flegrei, in the zeolitized ignimbrite (our LYT unit).

*Interpretation*

The localized distribution of this facies that passes laterally to a sintered, valley-ponding deposit excludes that it may be the product of a phreatomagmatic phase. The internal structure of accretionary lapilli reflects the way in which they formed^20^. They proposed that accretionary lapilli start to form within the coignimbrite ash plume (as ash pellets) and then settle into pyroclastic density currents, where they accrete successive concentric laminations. We suggest that accretionary lapilli settling from a phoenix could be incorporated in a pyroclastic current but would not produce a temperature drop so significant as to deposit unconsolidated products. The distribution in a well-defined topographic setting, parallel to the Apennine ridge, suggests that localized rainstorms may have affected the ash-rich PC^21^ dropping the PC temperature and promoting ash aggregation. The unconsolidated occurrence of this facies indicates that the emplacement temperature was below the zeolitization threshold^22^ (120°C), preventing the development of the lithification process. The ash aggregated around a fine pumice fragment, acting as a core, and then fell through the dilute PDC and accreted by successive concentric layers. This process was efficient but extremely localized, generating an overbank incoherent facies, rich in accretionary lapilli, while in few tens of metres, the valley-ponding facies shows its common sintered nature.

**REFERENCES**

1. Kneller, B.C. & Branney, M. J. Sustained high‐density turbidity currents and the deposition of thick massive sands. *Sedimentology* **42**(4), 607-616 (1995).
2. Vrolijk, P.J. & Southard, J.B. Experiments on rapid deposition of sand from high-velocity flows. *Geoscience Canada* **24**, 45-54 (1997).
3. Branney, M. & Kokelaar, P. Pyroclastic density currents and the sedimentation of ignimbrites. *Geol. Soc. London Memoir* **27,** 1-143 (2002).
4. Fedele, L., Scarpati, C., Sparice, D., Perrotta, A. & Laiena, F. A chemostratigraphic study of the Campanian Ignimbrite eruption (Campi Flegrei, Italy): insights on magma chamber withdrawal and deposit accumulation as revealed by compositionally zoned stratigraphic and facies framework. *J. Volcanol. Geotherm. Res*. **324**, 105–117 (2016).
5. Lowe, D.R. Suspended‐load fallout rate as an independent variable in the analysis of current structures. *Sedimentology*, **35**(5), 765-776 (1988).
6. Cole, P.D. & Scarpati, C. A facies interpretation of the eruption and emplacement mechanisms of the upper part of the Neapolitan Yellow Tuff, Campi Flegrei, southern Italy. *Bull. Volcanol.* **55,** 311-326 (1993).
7. Sulpizio, R., Dellino, P., Doronzo, D. M. & Sarocchi, D. Pyroclastic density currents: state of the art and perspectives. *J. Volcanol. Geotherm. Res.* **283,** 36-65 (2014).
8. Carrasco-Núñez, G. & Branney, M. J. Progressive assembly of a massive layer of ignimbrite with a normal-to-reverse compositional zoning: the Zaragoza ignimbrite of central Mexico. *Bull. Volcanol*. **68**(1), 3 (2005).
9. Scarpati, C., Sparice, D. & Perrotta, A. The ground layer of the Campanian Ignimbrite: an example of deposition from a dilute pyroclastic density current. *Bull. Volcanol.* **77**(11), 97 (2015).
10. Walker, G.P.L. Crystal concentration in ignimbrites. *Contr. Mineral. Petrol.* **36**(2), 135-146 (1972).
11. Scarpati, C., Sparice, D. & Perrotta, A. A crystal concentration method for calculating ignimbrite volume from distal ash-fall deposits and a reappraisal of the magnitude of the Campanian Ignimbrite. *J. Volcanol. Geotherm. Res.* **280,** 67-75 (2014).
12. Walker, G.P.L, Self, S. & Frogatt, P.C. The ground layer of the Taupo Ignimbrite: A striking example of sedimentation from a pyroclastic flow. *J. Volcanol. Geotherm. Res.* **10,** 1-11 (1981).
13. Sparks, R.S.J. *et al*. Ignimbrites of the Cerro Galan Caldera, NW Argentina. *J. Volcanol. Geotherm. Res.* **24**(3), 205-248 (1985).
14. Sulpizio, R., Mele, D., Dellino, P. & La Volpe, L. Deposits and physical properties of pyroclastic density currents during complex Subplinian eruptions: the AD 472 (Pollena) eruption of Somma-Vesuvius, Italy. *Sedimentology* **54**(3), 607-635 (2007).
15. Brand, B.D., Bendaña, S., Self, S. & Pollock, N. Topographic controls on pyroclastic density current dynamics: Insight from 18 May 1980 deposits at Mount St. Helens, Washington (USA). *J. Volcanol. Geoth. Res*. **321,** 1-17 (2016).
16. Douillet, G.A. *et al.* Pyroclastic dune bedforms: macroscale structures and lateral variations. Examples from the 2006 pyroclastic currents at Tungurahua (Ecuador). *Sedimentology*, **66**(5), 1531-1559 (2019).
17. Douillet, G.A. *et al*. Dune bedforms produced by dilute pyroclastic density currents from the August 2006 eruption of Tungurahua volcano, Ecuador. *Bull. Volcanol.* **75**(11), 762 (2013).
18. Perrotta A., Scarpati C., Giacomelli L. & Capozzi A.R. Proximal depositional facies from a caldera forming eruption: The Parata Grande Tuff at Ventotene island (Italy). *J. Volcanol. Geotherm. Res.* **71,** 207-228 (1996).
19. Smith, G. *et al.* A bedform phase diagram for dense granular currents. *Nature communications* **11**(1), <https://doi.org/10.1038/s41467-020-16657-z> (2020).
20. Brown, R. J. & Branney, M. J. Internal flow variations and diachronous sedimentation within extensive, sustained, density-stratified pyroclastic density currents flowing down gentle slopes, as revealed by the internal architectures of ignimbrites on Tenerife. *Bull. Volcanol.* **75**(7), 727 (2013).
21. Moore, J.G. & Melson, W.G. Nuees ardentes of the 1968 eruption of Mayon volcano, Philippines. *Bull. Volcanol.* **33,** 600-620 (1969).
22. Langella, A. *et al.* New insighs on the mineralogical facies distribution of Campanian Ignimbrite, a relevant Italian industrial material. *App. Clay Sci*. **72**, 55–73 (2013).
